# Phytocannabinoids and Cannabis-Based Products as Alternative Pharmacotherapy in Neurodegenerative Diseases: From Hypothesis to Clinical Practice

**DOI:** 10.3389/fncel.2022.917164

**Published:** 2022-05-30

**Authors:** Yolanda Paes-Colli, Andrey F. L. Aguiar, Alinny Rosendo Isaac, Bruna K. Ferreira, Raquel Maria P. Campos, Priscila Martins Pinheiro Trindade, Ricardo Augusto de Melo Reis, Luzia S. Sampaio

**Affiliations:** ^1^Instituto de Biofísica Carlos Chagas Filho (IBCCF), Centro de Ciências da Saúde, Universidade Federal do Rio de Janeiro, Rio de Janeiro, Brazil; ^2^Instituto de Bioquímica Médica Leopoldo De Meis (IBqM), Centro de Ciências da Saúde, Universidade Federal do Rio de Janeiro, Rio de Janeiro, Brazil

**Keywords:** Alzheimer’s disease, Parkinson’s disease, Multiple Sclerosis, Cannabis, CBD—cannabidiol, THC—tetrahydrocannabinol, endocannabinoid system (ECS)

## Abstract

Historically, Cannabis is one of the first plants to be domesticated and used in medicine, though only in the last years the amount of Cannabis-based products or medicines has increased worldwide. Previous preclinical studies and few published clinical trials have demonstrated the efficacy and safety of Cannabis-based medicines in humans. Indeed, Cannabis-related medicines are used to treat multiple pathological conditions, including neurodegenerative disorders. In clinical practice, Cannabis products have already been introduced to treatment regimens of Alzheimer’s disease, Parkinson’s disease and Multiple Sclerosis’s patients, and the mechanisms of action behind the reported improvement in the clinical outcome and disease progression are associated with their anti-inflammatory, immunosuppressive, antioxidant, and neuroprotective properties, due to the modulation of the endocannabinoid system. In this review, we describe the role played by the endocannabinoid system in the physiopathology of Alzheimer, Parkinson, and Multiple Sclerosis, mainly at the neuroimmunological level. We also discuss the evidence for the correlation between phytocannabinoids and their therapeutic effects in these disorders, thus describing the main clinical studies carried out so far on the therapeutic performance of Cannabis-based medicines.

## Introduction

Neurodegenerative diseases impact millions of people worldwide, affecting the wellbeing of patients and their relatives, besides social, economic and health burden due to disease management. In 2005, according to epidemiological data released by the World Health Organization (WHO), neurological diseases, such as Alzheimer’s Disease (AD), Parkinson’s Disease (PD), and Multiple Sclerosis (MS), when combined, corresponded to 1.0% of diseases that led to premature death and loss of years of life in consequence of a disability (World Health Organization [WHO], 2006). In addition, it is expected the increase in incidence of these diseases in the world population in the next decades, being estimated that 1.5% of worldwide deaths would occur as a consequence of AD, PD, and MS (World Health Organization [WHO], 2006).

Even though AD, PD, and MS have different physiopathological characteristics, such as diverse symptoms and clinical signs, besides specific age groups for patients affected by each of these neurodegenerative diseases, they also share some common aspects. First, none of these diseases have efficient long-term treatments, due to elevated side effects of current therapies and their progressive lessening of efficacy; secondly, they are associated with high social and economic impact because the disease progression demands extensive need of palliative care, which has great impact on the daily routine of patients and their caregivers (Wirdefeldt et al., 2011; Gauthier et al., 2021).

In the last decades, the endocannabinoid system (ECS) has emerged as a significant element to orchestrate physiological and physiopathological processes (Rodríguez de Fonseca et al., 2005; Stasiulewicz et al., 2020). Several preclinical and clinical studies have described an unbalance in ECS components in animal models and patients diagnosed with AD, PD, and MS (Cristino et al., 2020). Also, more recent studies focusing on animal models of neurodegenerative diseases showed that modulation of ECS is a valid alternative to improve animal’s conditions (Ramírez et al., 2005; García-Arencibia et al., 2007; Zhao et al., 2020; Reynoso-Moreno et al., 2021).

Based on scientific evidence, the use of Cannabis-based products or Cannabis-based medicine (CBM) has been growing among patients diagnosed with neurodegenerative diseases (Solimini et al., 2017). Most reports of clinical trials also describe significant improvement in disease-related primary and/or secondary symptoms, besides general improvement in life quality (Maroon and Bost, 2018; Milano, 2018). Even though there is still a lot to be uncovered in relation to CBM-mediated modulation of ECS, it is speculated that the improvement reported in patients’ wellbeing is mostly related to the antioxidant and anti-inflammatory properties of phytocannabinoids (Maayah et al., 2020), which have several targets in the so-called endocannabinoidome, whose concept comprehends an expansion of the previous ECS to include enzymes, receptors and secondary messengers regulated by cannabinoids (Ligresti et al., 2016; Cristino et al., 2020).

In this review, we describe the main findings concerning the involvement of ECS in AD, PD and MS in animal models, as well as outcomes reported in clinical trials. Furthermore, we focus on the use of CBM for treatment of these neurodegenerative diseases and the assessment of efficacy, safety, and tolerability of this therapeutic tool in humans.

## Endocannabinoid System

The ECS is classically formed by the two main specific cannabinoid receptors (CB1 and CB2), the endogenous cannabinoids, such as anandamide (AEA) and 2-arachidonoylglycerol (2-AG), and the enzymes responsible for their synthesis and degradation, for example monoacylglycerol lipase (MAGL) and fatty acid amide hydrolase (FAAH), which degrade 2-AG and AEA, respectively (Ligresti et al., 2016). This system is responsible for the regulation of several physiological processes in the Central Nervous System (CNS), endocrine, immune, gastrointestinal, and reproductive systems, among others (Di Marzo et al., 1998; Aizpurua-Olaizola et al., 2017). The endocannabinoids are mainly bioactive lipids derived from the cleavage of membrane fatty acids and phospholipids, and their levels can be modulated by diet, exercise and lifestyle (de Melo Reis et al., 2021; Isaac et al., 2021; Sihag and Di Marzo, 2022).

Studies about the structure and activity of cannabinoids extracted from *Cannabis sativa* have contributed to the development of synthetic cannabinoids, which allowed the discovery of the cannabinoid receptors and the identification of physiological functions modulated by this drug class (Howlett et al., 1990). After the discovery of cannabinoid receptors, studies led to endocannabinoid identification and their biosynthetic pathways, characterizing the complex ECS machinery.

The two main classes of endocannabinoids are N-acylethanolamines (NAEs) and 2-monoacylglycerols (2-MAGs). NAEs include arachidonoyl ethanolamide, also known as AEA, and endocannabinoid-like molecules such as palmitoylethanolamide (PEA) and oleoylethanolamide (OEA), which are mainly derived from the cleavage of N-acyl phosphatidylethanolamines (NAPE) by NAPE-specific phospholipase-D enzyme (NAPE-PLD) and degraded mainly by FAAH to produce fatty acids and ethanolamine. 2-AG is a 2-MAG endocannabinoid synthesized from fatty acids by the action of diacylglycerol lipase (DAGLα and DAGLβ) and degraded by MAGL, producing glycerol and arachidonic acid (AA) (Sihag and Di Marzo, 2022). There are also several non-canonical pathways by which these lipids can be synthesized and degraded (Cristino et al., 2020).

Several pharmacological studies revealed the existence of other receptors, beside CB1 and CB2, that are not selective to cannabinoids, but are also responsive to these molecules, such as the transient receptor potential channels of vanilloid type-1 (TRPV1) and transient receptor potential cation channel subfamily M member 8 (TRPM8) (Zygmunt et al., 1999; De Petrocellis et al., 2007), the orphans G-protein-coupled receptors (GPCR) 55, 119, and 18 (GPR55, GPR119, and GPR18), Peroxisome Proliferator-Activated Receptor Gamma (PPARγ), among others (Hájos et al., 2001; De Petrocellis and Di Marzo, 2009; Cristino et al., 2020; Campos et al., 2021).

## Phytocannabinoids and Cannabis-Based Medicine

Cannabis is one of the first plants to be historically used for medical, religious, and recreational purposes, dating back to 5,000 years ago (Li, 1974; Bonini et al., 2018). Cannabinol (CBN) was the first plant phytocannabinoid to be isolated in late nineteenth century, while the structures, stereochemistry and synthesis of cannabidiol (CBD) and Δ^9^-tetrahydrocannabinol (THC) were elucidated by Professor Raphael Mechoulam in the 1960s (Mechoulam and Hanus, 2000). Phytocannabinoids are produced in their acid form, being decarboxylated due to heating, light conditions and even storage duration (Pertwee, 2006). Thus far, more than 100 phytocannabinoids have already been described in *Cannabis sativa L.* Concerning activity on CB1 and CB2 receptors, THC and Δ^9^-tetrahydrocannabivarin (THCV) are able to act as their agonist and antagonist, respectively. There are also other phytocannabinoids, such as CBD, THCV, cannabigerol (CBG), cannabigerovarin (CBGV), and cannabidivarin (CBDV), that exert activity on components of the endocannabinoidome, for example in TRPV1 and TRPV2 receptors (Di Marzo and Piscitelli, 2015).

It is worth mentioning that CBD, besides modulating the receptors cited above, is also capable of modulating 5-HT_1A_, PPAR, α1β, and α1 glycine receptors and transient receptor potential channel of ankyrin type-1 (TRPA1) (Russo et al., 2005; Ahrens et al., 2009; De Petrocellis et al., 2011). This broad spectrum of action gives this molecule the potential to be used in a wide range of physiopathological events, which have been more intensely explored in the last years. Additionally to their effect on endocannabinoidome receptors, CBD also inhibits AEA reuptake and FAAH activity, which is the main enzyme responsible for AEA hydrolysis, thus CBD treatment is able of increasing AEA levels (Di Marzo and Piscitelli, 2015; Campos et al., 2021).

Since their discovery, THC and CBD remain the two most studied phytocannabinoids for therapeutic application. THC is majorly associated with the modulation of pain-related stimuli, sedation, appetite and mood, besides action as bronchodilator and antioxidant with neuroprotective and anti-inflammatory potential (Williams et al., 1976; Russo and Marcu, 2017). THC agonist activity on CB1 induces four behavioral traits underlying its psychotropic effect in animal models, known as cannabinoid tetrad: hypolocomotion, hypothermia, catalepsy, and antinociception, while its action on CB2 is correlated with anti-inflammatory properties and pain relief (Russo and Marcu, 2017). Even though CBD is not a direct agonist of cannabinoid receptors and acts as a negative allosteric modulator of CB1, the absence of psychotropic effect following its administration and its anti-inflammatory and immunosuppressive activity due to ECS modulation increases the possibility of medical application of this molecule in terms of several clinical conditions, from pediatric to adult patients (Di Marzo and Piscitelli, 2015; Russo and Marcu, 2017; Sampson, 2021).

Even though *Cannabis sativa L.* had been used for therapeutic purposes since Antiquity, it was only in the early 2000s that pharmaceutical grade CBM were developed and applied to treatment of several diseases. The first CBM approved by regulatory agencies consisted of an oromucosal spray with 1:1 ratio of CBD and THC, commonly prescribed to MS patients in order to alleviate spasticity and extensively evaluated in terms of pharmacokinetics and pharmacodynamics (Lucas et al., 2018; Cristino et al., 2020). Different formulations and routes of administration for phytocannabinoids are currently available, although CBM are majorly found as either oils or tinctures for oromucosal administration, which allows quick absorption and high plasmatic levels of THC and CBD (Lucas et al., 2018; Mohamed et al., 2022). Subsequent to oromucosal administration of CBM, the concentration of CBD and THC reaches its peak within the first hour and doses of THC 5.4 mg and CBD 5.0 mg show half-life of 1.94 and 5.28 h, respectively (Stott et al., 2013).

Apart from THC and CBD metabolization by hepatic microsomal enzymes, cytochrome P450 (CYP), these phytocannabinoids are also able to modulate the activity of some CYP isoforms. THC hydroxylation is conducted by CYP2C9, leading to the active metabolite 11-hydroxy-THC (11-OH-THC), which may be further hydroxylated by CYP2C9 and results in the biologically inactive 11-nor-9-carboxy-THC (THC-COOH). In addition to this classic pathway, it has already been described that THC may be also metabolized by CYP2C19 and CYP3A4. Concerning CBD metabolism, it occurs preferentially by CYP2C19, resulting in 7-hydroxy-CBD (7-OH-CBD), although it can be used as substrate for CYP3A4, CYP1A1, CYP1A2, CYP2C9, and CYP2D6 (Lucas et al., 2018; Nasrin et al., 2021). Besides the metabolism of phytocannabinoids mediated by CYP activity, *in vitro* assays have shown that THC and CBD are able to inhibit CYP isoforms. For example, THC inhibits CYP1A1, CYP2A6, and CYP2C9 activity, while CBD suppresses enzymes such as CYP1A1, CYP2A6, CYP2B6, CYP2C9, CYP2D6, CYP2C19, and CYP3A4 (Cox et al., 2019; Doohan et al., 2021; Nasrin et al., 2021).

Considering the pharmacodynamics of CBM, THC acts as an agonist of cannabinoid receptors and shows additional effect as agonist of other receptors described as part of the endocannabinoidome, such as TRPV1 and GPR55. On the other hand, CBD acts on a vast repertoire of receptors and intermediate mediators of the endocannabinoidome; for example, it can play the role of agonist of TRPV1, TRPV4, 5-HT1A, PPARγ receptor, antagonist of TRPM8, and GPR55, besides inhibiting FAAH activity and promoting negative allosteric modulation of CB1 (Cristino et al., 2020; Sampson, 2021).

These data related to the modulation of physiological systems by phytocannabinoids raise awareness toward the therapeutic use of THC, CBD, and other minor phytocannabinoids. Thus, the activity of these compounds regulating inflammatory processes, immune response, and neuronal activity supports their use as either adjunctive therapy or central agents for controlling the development and prognosis of neurodegenerative diseases.

## Alzheimer’s Disease

### Pathological Properties of Alzheimer’s Disease

**FIGURE 1:**
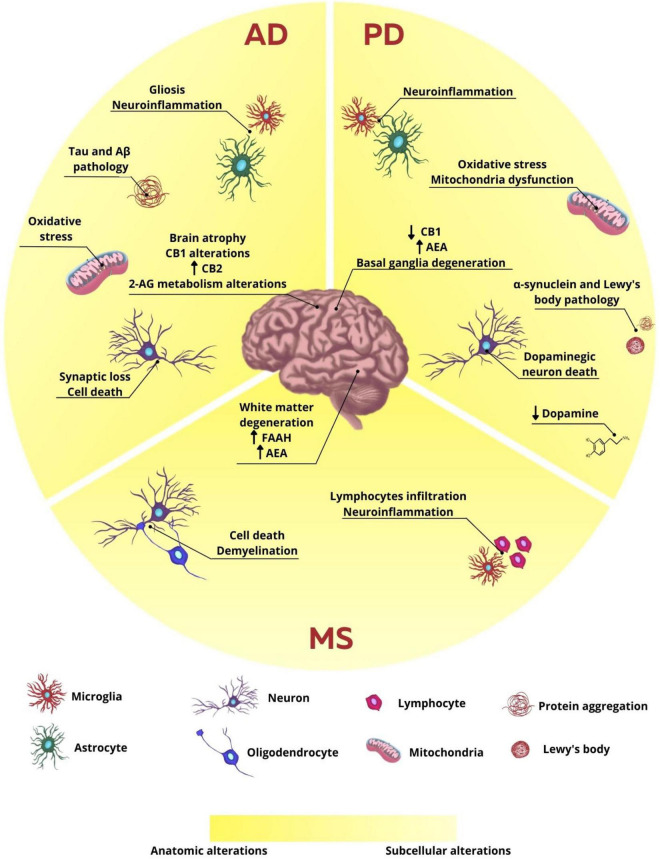
Anatomic and subcellular alterations in neurodegenerative diseases. In AD, the characteristic subcellular alteration is the intracellular aggregates formed by hyperphosphorylated tau protein and extracellular plaques of β-amyloid protein, neuroinflammation associated with gliosis, oxidative stress, synaptic loss, and cell death. The endocannabinoid system is also altered with alteration in CB1, CB2, and 2-AG expression and production, respectively. These cellular changes are related to brain atrophy observed in patients and animals. In PD, the main cellular observation is the loss of dopaminergic neurons and consequent decrease in dopamine release. There is also protein aggregation of α-synuclein and the formation of Lewy bodies, neuroinflammation, and oxidative stress, which leads to basal ganglia degeneration. CB1 expression is reduced and AEA levels are increased. In MS, however, neuroinflammation is the main characteristic of the disease, once lymphocytes infiltrate the brain and microglia reaction. There is also cell death and demyelination, leading to white matter degeneration. FAAH expression and AEA levels are increased.

AD is the most prevalent neurodegenerative disease in the world, affecting around 40–50 million people globally, and is the main cause of dementia, a general term used to describe memory loss and cognitive impairment in individuals over 65 years old. AD is characterized by the presence of extracellular deposits of β-amyloid protein aggregates (Aβ), forming amyloid plaques, and by the presence of intracellular neurofibrillary tangles containing hyperphosphorylated tau protein (Pooler et al., 2014; Spires-Jones and Hyman, 2014; Liu et al., 2019; Long and Holtzman, 2019; Knopman et al., 2021). Although the etiology of AD is not totally understood, it is known that several genetic and environmental factors are involved with the emergence of this disease. Mutations in some genes, such as amyloid precursor protein (APP) and presenilin-1 (PSEN1) and presenilin-2 (PSEN2) proteins, related to the production of β-amyloid peptides, are directly associated to the development of familiar AD, distinguished by early onset. Therefore, mutations in these genes, that correspond to 1–5% of all cases, are considered determinant to the emergence of this disease (Selkoe, 2011; Knopman et al., 2021). On the other hand, mutations or polymorphisms in other genes that code proteins such as apolipoprotein E (ApoE) are considered risk factors to the development of late onset AD. Polymorphism in the allele 4 of ApoE (ApoE-e4), for example, appears in about 40–65% of the individuals diagnosed with AD and it is related to the unbalance in Aβ clearance, leading to its accumulation and aggregation (Long and Holtzman, 2019; Yamazaki et al., 2019). Non-genetic and environmental factors, such as exposure to toxins/viruses, head trauma, cardiovascular diseases, diabetes, and sedentary lifestyle may also be associated with a higher probability of AD development (Selkoe, 2011; Karch and Goate, 2015; Armstrong, 2019). Therefore, it is believed that alterations in Aβ metabolism and dysfunctional tau protein phosphorylation may be the main causes to the emergence of AD, leading to altered synaptic signaling, activation of glial inflammatory responses, alterations in ionic homeostasis and oxidative stress, as well as activation of intracellular pathways in response to stress. Thus, the homeostatic alterations produced by these factors lead to neuronal damage and consequent cell death, contributing to the already known cognitive and memory deficits (Sperling et al., 2011; Heneka et al., 2015; Masters et al., 2015; Ding et al., 2019; Hampel et al., 2021; Figure 1).

Despite the growing scientific advances regarding the comprehension of cellular and molecular mechanisms underlying AD physiopathology, this disease still has no cure. This way, the pharmacotherapy used in the clinic aims to reduce some of the symptoms, though not preventing the progression of the disease and the considerable decrease in life quality of both patients and caretakers.

### Endocannabinoid System Alterations in the Context of Alzheimer’s Disease

Although there are few conflicting studies, research involving animal models and post-mortem brain tissue of diagnosed patients have already shown that elements of the ECS may be altered in this pathology (Figure 1). Some studies show, for example, that the expression of CB1 receptors in brain areas such as cortex, hippocampus and basal ganglia is decreased in AD patients (Westlake et al., 1994; Solas et al., 2013), while other studies show no difference in the expression of this receptor in cortex and hippocampus (Lee et al., 2010; Ahmad et al., 2014). On the other hand, studies appear to be more consistent regarding the increase in CB2 receptor expression in AD, which may be correlated to the microglial alteration found in this pathology (Benito et al., 2003; Solas et al., 2013; Basavarajappa et al., 2017; Hopperton et al., 2018). Evidence also shows that 2-AG levels were reduced in the plasma of AD individuals, besides its correlation to cognitive decline presented by these patients, suggesting a possible protective role of 2-AG (Altamura et al., 2015). Additionally, the levels of DAGL and MAGL, enzymes responsible by the canonical pathway of synthesis and degradation of 2-AG, respectively, were increased in the hippocampus of post-mortem AD patients in Braak VI stage, indicating that 2-AG metabolism might be altered according to the stage of the disease (Mulder et al., 2011).

### Endocannabinoid System Modulation as a Treatment for Alzheimer’s Disease

The ECS modulation has been studied as an alternative to AD treatment (Mulder et al., 2011; Manuel et al., 2014). Preclinical studies performed *in vitro* and *in vivo* using synthetic drugs to modulate the ECS have already shown positive results regarding the reduction of Aβ plaque deposition and tau phosphorylation, improvement in the cognitive performance and reduction of glial activation and neuroinflammation (Ramírez et al., 2005; Chen et al., 2011, 2019, 2012; Aso et al., 2012; Murphy et al., 2012; Zhang and Chen, 2018; Zhao et al., 2020; Grieco et al., 2021). On the other hand, the use of synthetic compounds, such as THC analogs, in clinical studies promoted an improvement only regarding aggressiveness and agitation in patients, not showing effectiveness in terms of primary symptoms of AD (Passmore, 2008; Woodward et al., 2014; Herrmann et al., 2019).

**FIGURE 2:**
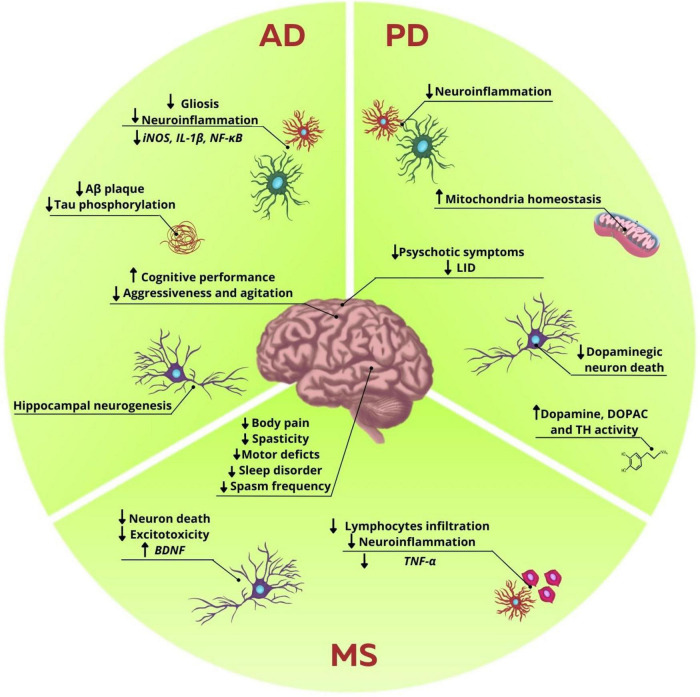
Influence of endocannabinoid system modulation in neurodegenerative diseases disturbances. In AD, ECS modulation reduces Aβ plaques and tau phosphorylation. It was also observed a decrease in gliosis and neuroinflammation, marked by the reduction in proinflammatory markers, such as iNOS, IL-1β, and NF-κB. Hippocampal neurogenesis was also observed. There is an increase in the cognitive performance and decrease of agitation and aggressiveness in patients. In PD, studies showed a decrease in neuroinflammation and in dopaminergic neuron death, while enhancing the levels of dopamine, DOPAC and TH activity. It was also observed an increase in mitochondrial homeostasis, and decrease of neuroinflammation. Behavior improvements are reduction in psychotic symptoms and LID. In MS excitotoxicity and neuron death are decreased, while there is an increase in BDNF levels. Neuroinflammation is also decreased with the reduction in lymphocytes infiltration and release of proinflammatory cytokines, such as TNF-α. There are several benefits regarding secondary symptoms, such as decrease in body pain, motor deficits, spasticity, sleep disorder, and spasms frequency, thus collaborating in the improvement of the patients’ life quality.

Phytocannabinoids have also been used in preclinical and clinical trials. In SH-SY5Y cell lineage transfected with APP (695), the treatment with CBD in several concentrations (10^–9^–10^–6^ M) decreased the content of APP and Aβ42 through activation of the PPARγ receptors (Scuderi et al., 2014). In rats injected with intrahippocampal Aβ, the treatment with CBD (10 mg/kg, i.p.) for 15 days led to the decrease in neuroinflammation and increase of hippocampal neurogenesis. Interestingly, these effects were abolished after administration of GW9662 (a PPARγ antagonist; 1 mg/kg, i.p.), reinforcing the involvement of this receptor in the beneficial effects following CBD administration (Esposito et al., 2011). Later, it has been shown that the treatment with CBD 2.5 or 10 mg/kg, i.p. during 7 days promoted a dose-dependent decrease in the iNOS, GFAP, and IL-1β expressions in the hippocampus of C57BL/6J mice injected with intrahippocampal Aβ (Esposito et al., 2007; Figure 2). Additionally, PC12 cultures incubated for 36 h with Aβ1-42 and treated with CBD (10^–6^–10^–4^ M) also had reduction in p38 MAPK phosphorylation, as well as decrease in the activity of NF-κB transcription factor, suggesting another mechanism of action to the protective effects observed with the use of this phytocannabinoid (Esposito et al., 2006).

Moreover, the synergic role of THC and CBD in animal models of AD has also been evaluated. In 6 months old APP/PS1 mice, the neuroprotective role of the treatment with isolated THC (0.75 mg/kg, i.p.), CBD (0.75 mg/kg, i.p.), or their combination for 5 weeks was assessed. Even though animals treated with either isolated phytocannabinoids or their combination showed improvement in tests designed to assess memory and learning, such as the novel object recognition and active avoidance, only animals that received THC + CBD had a decrease in soluble Aβ42 levels and gliosis in cerebral cortex (Aso et al., 2015). On the other hand, when the same experimental design was performed with 12-months old mice, the positive results observed regarding Aβ42 levels and gliosis were lost, suggesting that the mechanisms of action mediated by these phytocannabinoids might be more effective at initial stages of AD (Aso et al., 2016; Figure 2).

Another important molecular feature found in neurodegenerative diseases is the failure in protein homeostasis mechanisms, resulting in undesirable aggregation of misfolded proteins. In this context, CBD has exerted its protective effect over several signaling cascades involved with proteostasis, consequently reducing oxidative stress in cells (Dash et al., 2021). It has been demonstrated that CBD is able to downregulate genes involved in development of AD in gingiva derived mesenchymal stem cells (GMSCs), more precisely in secretases responsible for Aβ generation and kinases involved in tau protein phosphorylation. It was also shown that CBD can upregulate the expression of several heat shock proteins (HSP) and the activity of ubiquitin systems, responsible to maintain proteostasis (Libro et al., 2017).

Regarding clinical studies using phytocannabinoids, the data are still feeble. However, studies show improvements in some important secondary symptoms related to AD. In a randomized controlled study with 11 patients diagnosed with AD or vascular dementia, the outcome of treatment with THC 1.5 mg, orally administered three times a day for 3 weeks, was evaluated. In this study, patients did not show improvements in the Neuropsychiatric Inventory (NPI) scale, therefore no amelioration of agitation, life quality aspects and performance in daily activities was reported (van den Elsen et al., 2015). Moreover, the same group showed that THC in the same dosage, twice a day, did not promote adverse effects regarding balance and gait of patients (van den Elsen et al., 2017). On the other hand, in an open-label pilot study with 11 AD patients, THC 2.5–5 mg twice a day for 4 weeks promoted improvement in the Clinical Global Impression (CGI) scale, as well as reduction in aggressiveness/agitation, apathy, irritability, and sleep disturbances, assessed by NPI. However, there was no effect regarding MMSE (Mini Mental State Examination) scores (Shelef et al., 2016).

Another pilot study conducted in a nursing home in Switzerland showed that the administration of CBM, with known THC:CBD concentrations (1:2 ratio) decreased the use of other psychotropic drugs used in the treatment of dementia in women with AD, besides improving behavioral and psychological symptoms of dementia. The CBM was administered with food to facilitate the intake, and the minimum dosage used was 7 mg of THC and 14 mg of CBD, while the maximum was 13 mg of THC and 26 mg of CBD per day, during the course of 2 months. It was reported improvement in neuropsychiatric and motor scores and in aggressiveness. The feedback offered by nurses and relatives was positive, when taking into consideration the noticeable reduction in aggressive behaviors, such as screaming and irritability, and motor issues, such as muscle stiffness, that collectively disturbed the life quality of other patients, staff and caregivers (Broers et al., 2019; Figure 2).

Recently, an extensive systematic review evaluating the use of phytocannabinoids, especially THC, in the treatment of AD, showed that this compound is effective in improving some secondary symptoms of this pathology, but not in aspects regarding memory deficits and cognition (Bosnjak Kuharic et al., 2021). Although in preclinical studies CBD has shown promising effects in controlling the characteristic neuroinflammation associated with AD models and behavioral improvements, there are still no clinical studies evaluating positive effects of CBD in the control of AD symptoms in patients.

In summary, the need for more studies using phytocannabinoids in different therapeutic dosages and treatment regimens is notorious, in order to better understand the effect of these compounds in AD, as well as to establish standardized therapies with phytocannabinoids.

## Parkinson’s Disease

### Pathological Properties of Parkinson’s Disease

PD is considered the second most prevalent neurodegenerative disease, only behind Alzheimer’s disease. It is estimated that about 1–2% of the population between 65 and 70 years old will present the diagnosis and, with the increase of life expectancy, it is believed that about 9 million people might present the disease in the next 10 years (Wirdefeldt et al., 2011; Tysnes and Storstein, 2017). One of the main PD symptoms is the motor disturbance characterized by bradykinesia, that consists in the presence of stiffness, tremors and slowing down of voluntary movements (Larsen et al., 1994). As the disease develops, other symptoms can appear, such as: cognitive alterations, depression, and anxiety (Lees and Smith, 1983; Schrag and Taddei, 2017). Additionally to the motor, cognitive and psychiatric damages, sensorial, autonomic, and sleep disturbances can develop in parkinsonian patients, most of the time manifesting years before the appearance of motor alterations (Chaudhuri K. R. et al., 2006; Sauerbier et al., 2017). These symptoms result from a series of molecular alterations that occur gradually during the disease.

At the cellular level, PD consists in the progressive death of dopaminergic neurons found in substantia nigra *pars compacta* and consequent loss of projections to the striatum, which is responsible for most of the motor deficit observed in patients (Dauer and Przedborski, 2003; Olanow et al., 2009). This neurodegenerative process results in the depletion of dopamine, promoting the dysfunction of important pathways related to the control of voluntary movements, involving the basal ganglia, cerebral cortex, thalamus, and brainstem (Alexander and Crutcher, 1990; Dauer and Przedborski, 2003; Olanow et al., 2009). The degeneration of dopaminergic neurons results from a series of dysfunctions intertwined, promoting a disbalance in cellular homeostasis and production of trophic factors. Among the processes involved are alterations in proteostasis, oxidative stress, mitochondrial damage, inflammation, and apoptosis (Olanow et al., 2009; Winklhofer and Haass, 2010; Stojkovska et al., 2015; Guo et al., 2018; Figure 1). The etiology of PD has not been clarified yet. However, it is suggested that its development might be related to environmental factors (Olanow et al., 2009; Ascherio and Schwarzschild, 2016), such as exposure to toxins and lifestyle, and genetic factors, such as mutations in genes like PARK1/4, PARK2, and PINK1 (Winklhofer and Haass, 2010; Ali et al., 2011; Riederer et al., 2019). Another important physiological feature in PD is the presence of Lewy bodies in substantia nigra, structures consisting of cytoplasmic inclusions mainly composed by the accumulation of α-synuclein aggregates, closely related to multiple neurodegenerative processes (Hurtig et al., 2000; Riederer et al., 2019).

To date there is no pharmacological treatment to prevent the progression of PD, with the control of the main symptoms being the protocol used. The main pharmacological treatment used in PD patients is levodopa, administered with peripheral dopa decarboxylase inhibitors, such as benserazide or carbidopa, due to its rapid peripheral metabolization. The synergic effect between these drugs allows a greater availability of levodopa to the brain, besides reducing the adverse effects related to the increase in peripheral dopamine. After a certain period of treatment, other pharmacological interventions may be necessary to maintain the dopaminergic levels and act over the other non-motor symptoms (Connolly and Lang, 2014). Because of the progressive neurodegeneration, the fluctuations in the response with levodopa treatment, referred to as “on-off,” become more frequent. There are “on” periods, when the drug exerts its effect over motor control, and “off” periods, when the clinical effects cease and motor symptoms reappear (Poewe et al., 2017). Consequently, patients that are chronically users of levodopa may develop dyskinesia—a collateral effect known as levodopa-induced dyskinesia (LID)—in the “on” period. The mechanisms to the development of LID are complex and, besides the strong dopaminergic component, there is also the involvement of glutamatergic, serotoninergic and endocannabinoid systems, neuroinflammatory mechanisms, among others (Zheng and Zhang, 2021). Several scientific studies have brought even more understanding over the mechanisms underlying PD, allowing the development of new strategies to treat it. In this way, several antioxidants, anti-inflammatory, and nutraceuticals have been indicated (Hang et al., 2016; Sarkar et al., 2016). Besides that, current adjuvant pharmacotherapies try to attenuate the disease’s symptoms without developing LID, such as anticholinergic drugs, dopaminergic agonists, and amantadine (Connolly and Lang, 2014).

### Endocannabinoid System Alterations in the Context of Parkinson’s Disease

Scientific evidence obtained in animal models of PD and patients show that there are alterations in components of the ECS in PD pathology. Using the experimental model of 6-hydroxydopamine (6-OHDA) injection into the substantia nigra of rats, it was observed an increasing in AEA levels in the striatum of lesioned animals, while observing a decreasing in the activity of the AEA membrane transporter, as well as the endocannabinoid degradation enzyme FAAH (Figure 1). It was also observed that the inhibition of this enzyme promoted a decrease in the glutamatergic activity in the striatum of animals injected with 6-OHDA. Other studies showed in PD models that the pharmacological increase in the endocannabinoid levels and/or direct activation of cannabinoid receptors was capable of reducing the death of dopaminergic neurons, recovering the dopamine levels and the activity of the enzyme tyrosine hydroxylase, as well as improving motor deficits and neuroinflammatory parameters (García-Arencibia et al., 2007; Price et al., 2009; Fernández-Suárez et al., 2014; Mounsey et al., 2015). These data suggest that the modulation of this system’s activity might be an interesting strategy to regulate the excessive excitability in the circuits connected to the basal ganglia in PD (Gubellini et al., 2002). To reinforce the relationship between the ECS alterations and PD, the analysis of the cerebrospinal fluid of patients showed an increase of almost twofold in the AEA levels (Pisani et al., 2005). Besides, a decrease in the expression of CB1 mRNA was detected in the striatum and globus pallidus external of post-mortem samples of individuals with PD (Hurley et al., 2003; Figure 1).

### Endocannabinoid System Modulation as a Treatment for Parkinson’s Disease

Regarding the physiological control of voluntary movements, the modulatory effects of the ECS are well established in the literature, mainly through the regulation of neurotransmission in structures of basal ganglia, such as striatum, globus pallidus external and internal, and substantia nigra. Moreover, the activation of cannabinoid receptors in subpopulations of GABAergic and glutamatergic neurons located in these regions also modulate the dopaminergic signaling, specially through interaction between CB1 receptors and dopaminergic receptors D1 and D2 (Benarroch, 2007; Bassi et al., 2017).

Nowadays, some preclinical and clinical studies try to unravel the mechanisms underlying the benefits that cannabinoids and CBM may have on the symptoms of PD. In this context, the neuroprotective effects of THC and CBD, as well as the possible action pathways of these molecules, have already been observed in toxicity models *in vitro*. In the cell lineage SH-SY5Y of human neuroblastoma incubated with MPP + toxin, the treatment with THC 10 μM increased the viability of these cells. Besides, THC also decreased cell death in response to other toxins, such as paraquat (inductor of oxidative stress) and lactacystin (inhibitor of the proteasome-ubiquitin system). Interestingly, these neuroprotector effects were not mediated by the activation of CB1 receptors, but PPARγ and its consequent involvement in regulating of redox homeostasis (Carroll et al., 2012). Additionally, the same group showed that the effects promoted by THC were related to prevention in mitochondrial mass reduction through increasing in PPARγ coactivator 1α (PGC-1α) expression, involved with metabolism regulation and mitochondrial biogenesis, as well as preventing reduction of the mitochondrial transcription factor A levels (TFAM), a protein involved in replication of mtDNA. This evidence highlights the protective role of THC through modulation of mitochondrial homeostasis (Zeissler et al., 2016). Cultures treated with CBD 10 μM for 48 h showed increased cell viability through activation of CB2 and TRPV1 receptors (Gugliandolo et al., 2020). Additionally, in another study using *in vitro* toxicity with MPP+, using PC12 cells, it was demonstrated that the treatment with CBD 1 μM for 24 h increased cell viability and differentiation, as well as a higher expression of GAP-43 protein, associated to neurite growth, and proteins associated to synaptic vesicles, such as synaptophysin and synapsin I (Santos et al., 2015; Figure 2).

The use of phytocannabinoids has also shown positive results in murine models of PD. With the 6-OHDA model, a consecutive treatment with isolated CBD (3 mg/kg/day, intraperitoneally) or THC (3 mg/kg/day, intraperitoneally) in rats showed a recovery in dopamine and DOPAC levels and tyrosine hydroxylase activity, both in substantia nigra and striatum (Lastres-Becker et al., 2005; Figure 2). Two years later, using the same isolated CBD treatment, the same group showed that the neuroprotective effects were only efficient if the treatment was initiated concurrently with the lesion. In this study, it was still shown that CBD promoted increase in the expression of mRNA of the enzyme Cu,Zn-Superoxide Dismutase (SOD1), suggesting that this compound might act over antioxidant pathways (García-Arencibia et al., 2007).

Other Cannabis components, besides CBD and THC, were also used as therapeutic strategies in experimental models of PD. β-caryophyllene, a terpene with cannabimimetic properties for acting as a CB2 agonist, showed antioxidant and anti-inflammatory effects, thus reducing the death of dopaminergic neurons in substantia nigra and striatum in a PD model induced by rotenone (Ojha et al., 2016). Besides, THCV, a minor phytocannabinoid, has also shown neuroprotective effects in a model induced by 6-OHDA (García et al., 2011). An important problem to be considered in the current treatment of PD concerns the development of LID. This way, studies testing the effects of phytocannabinoids in this aspect are extremely relevant in the clinic. In animal models of LID, for example, the use of CBD and THCV showed positive results, decreasing stereotyped movements as well as proinflammatory markers (Figure 2). Regarding CBD, these effects were mainly attributed to their action over PPARγ and TRPV1 receptors (Dos-Santos-Pereira et al., 2016; Sonego et al., 2018; Espadas et al., 2020).

Clinical trials using phytocannabinoids with parkinsonian patients, besides presenting variable effects, point to the promising effects in the use of these phytocannabinoids and CBM in the disease’s symptoms. An open pilot study aimed to evaluate the efficacy of isolated CBD in patients diagnosed with PD presenting secondary psychotic symptoms. The use of crescent doses of isolated CBD (150–400 mg/day) for 4 weeks showed a progressive improvement in the psychosis symptoms related to the disease, but not motor parameters (Zuardi et al., 2009; Figure 2). Similarly, the same group showed, in a double-blind exploratory study, that the treatment with isolated CBD in capsules (300 mg/day) during 6 weeks induced improvement in daily activities and emotional wellness of patients when compared to those treated with placebo. However, changes related to motor behavior were not reported (Chagas et al., 2014).

In a crossed, randomized, double-blind, placebo-controlled study, Carroll et al. (2004) assessed the potential effect of a CBM (Cannabis extract containing a THC:CBD proportion of about 2:1, each capsule containing 2.5 mg of THC and 1.25 mg of CBD) in PD patients that presented LID. Although the authors had observed that oral treatment was well tolerated, the patients did not show relief in parkinsonian symptoms. Besides, despite the double-blind design, 71% of the patients correctly identified their respective treatment group. Additionally, it was not observed any effect of the use of CBM over the LID symptoms, as evaluated by the UPDRS questionnaire (Unified Parkinson’s Disease Rating Scale) and by Rush dyskinesia evaluation scale. There was also no improvement in any measures of secondary results, such as other motor parameters of UPDRS score, PDQ-39, pain or sleep quality (Carroll et al., 2004).

This way, it is observed that clinical studies involving PD and CBM or isolated phytocannabinoids are also very incipient and with few conclusive data. Nevertheless, the use of CBM is promising, especially when considering all previous evidence involving basic science with synthetic drugs or phytocannabinoids. Moreover, a relevant factor regarding the variety of results obtained in clinical research points to the variability in establishing and standardizing the time of treatment, the dosage of CBM or isolated components, and administration routes.

## Multiple Sclerosis

### Pathological Properties of Multiple Sclerosis

MS is a chronic autoimmune and progressive neurological disorder that affects more than 3 million people worldwide and is considered the most prevalent neurodegenerative disease in young adults (Reich et al., 2018). Disturbances in the neurovascular unit allow the infiltration of auto-reactive T cells into the CNS, which is facilitated by the activity of endothelial adhesion molecules (ICAM-1 and VCAM-1) and matrix metalloproteinases (MMP-2 and MMP-9), therefore promoting the degradation of myelin sheath and multifocal demyelinating lesions, mostly in the white matter, due to several immune-mediated mechanisms already postulated, such as cytokine release, cytotoxic attack of CD8^+^ T cells and macrophage-induced digestion of surface myelin antigens (Podbielska et al., 2021; Figure 1).

Even though the precise cause of MS may not be fully elucidated, several risk factors that could lead to disease onset have already been identified. Among environmental triggers for MS, infection by Epstein-Barr virus, vitamin D deficiency, obesity, tobacco consumption and exposure to toxins are highlighted, while genome-wide association studies identified more than 200 gene variants that could lead to elevated risk of MS development, such as the human leukocyte antigen DRB1*1501 haplotype (Reich et al., 2018).

The clinical progression of MS is classified according to the stages of progression and/or improvement of MS severity. Most patients experience a relapsing course of MS progression (relapsing-remitting multiple sclerosis, RRMS), which could become progressive in later stages, showing signs of steadily worsening neurological damage (secondary progressive multiple sclerosis, SPMS). In other cases, which accounts for the minority of patients, the disease gets progressive since the onset, with no relapsing stages (primary progressive multiple sclerosis, PPMS) (Absinta et al., 2020). Despite the different etiologies of MS, clinical manifestations may include optic neuritis, myelitis, motor and vestibular impairment, paroxysms, Uhthoff’s phenomenon, fatigue, and cognitive disorders (Frohman E. M., 2003).

In the pathogenesis of MS, glutamate-mediated excitotoxicity may greatly contribute to disease progression due to induced neuronal death, axonal loss and demyelination (Macrez et al., 2016). Not only neurons are responsible for increased release of glutamate in synapses, but also other cell types may contribute to the elevated level of this neurotransmitter. In the context of infiltration of peripheral immune cells into the CNS, monocytes, macrophages and dendritic cells, besides microglia, are able to release glutamate through the cysteine/glutamate antiporter Xc^–^ activity (Pacheco et al., 2006; Pampliega et al., 2011; Evonuk et al., 2015). The excessive glutamate may also induce AMPA-mediated excitotoxic cell death of oligodendrocytes, NMDA-mediated damage to myelin integrity, and overexpression of sodium-channel subunits Na_*v*_1.2 and Na_*v*_1.6 the white matter, resulting in Na^+^ accumulation in axons (Stys et al., 1993; Domercq et al., 2005; Young et al., 2008; Micu et al., 2016).

Furthermore, glial cells can exert a significant effect on neuroinflammatory-induced changes in CNS, which leads to aggravation of MS. Following injure in the neurovascular unit, T-cells infiltrate the CNS and release proinflammatory cytokines, such as TNF-α, IL-17, and IFN-γ, which in turn induce the activation of astrocytes and microglia (Rothhammer and Quintana, 2015). Excessive microglial activation is correlated to elevated release of reactive oxygen and nitrogen species (ROS and RNS, respectively) in MS and is particularly harmful to oligodendrocytes precursor cells (OPC) due to their low level of antioxidant enzymes and antiapoptotic proteins (Butts et al., 2008; Gray et al., 2008; Fischer et al., 2012; Figure 1).

### Endocannabinoid System Alterations in the Context of Multiple Sclerosis

The activation of CB1 and CB2 receptors is essential to control pre-synaptic release of glutamate in excitatory synapses and to modulate the release of proinflammatory cytokines produced by glial cells, respectively, contributing to mitigate excitotoxicity and neuroinflammation (Ligresti et al., 2016). In the pathological context of MS, imbalance of ECS components and cannabinoid-mediated signaling pathways may account for the perpetration of abnormal insults to homeostasis and consequent MS symptomatology (Rossi et al., 2010).

In animal model of experimental autoimmune encephalomyelitis (EAE), AEA, and 2-AG levels have been found downregulated in striatum, midbrain, brainstem, hippocampus, and cerebral cortex, which could be reverted by administration of AM404, an inhibitor of FAAH enzyme (Cabranes et al., 2005). Moreover, lower levels of endocannabinoids were also reported in cerebrospinal fluid of MS patients, highly correlated to disease subtype and relapse stage (Di Filippo et al., 2008). On the other hand, increased levels of AEA have been reported in cerebrospinal fluid, inflammatory lesions, lymphocytes and plasma of MS patients, despite lack of change in 2-AG levels (Eljaschewitsch et al., 2006; Centonze et al., 2007; Jean-Gilles et al., 2009; Figure 1).

Besides altered levels of endocannabinoids, the expression of FAAH has been reported as upregulated in MS plaques obtained from brain tissue of human patients, which may seem controversial when compared to AEA concentration in other tissues and/or fluids (Benito et al., 2007; Figure 1). Usually, delivery route, tissue origin, cannabinoid pharmacokinetics and pharmacodynamics, outcome measurement and other bias are accounted as responsible for diverging observations.

### Endocannabinoid System Modulation as Treatment for Multiple Sclerosis

Cannabinoids have been used as a tool to better elucidate *in vitro* and *in vivo* the potential role of ECS in reducing excitotoxicity and neuroinflammation. It has been reported that cannabinoid receptor agonists exert neuroprotective effects in mixed cortical cultures exposed to AMPA and NMDA, therefore reducing neuronal death (Docagne et al., 2007; Loría et al., 2010). Additionally, the CB1-mediated signaling is essential for tremor and spasticity control in animal model of chronic relapsing experimental autoimmune encephalomyelitis (CREAE) and EAE in CB1 knockout mice (Baker et al., 2000; Pryce and Baker, 2007). Besides, the dual activation of CB1 and CB2 by WIN 55,212-2, a cannabinoid agonist, improves the clinical score shown by animals, reduces inflammation and restores tolerance to self-myelin antigen (Arevalo-Martin et al., 2012), while WOBE437, an inhibitor of eCB reuptake, reduces MS severity and infiltration of immune cells into the CNS (Reynoso-Moreno et al., 2021).

Considering the use of phytocannabinoids in animal models of MS, it has been described that CBD is able to attenuate infiltration of T cells into the spinal cord, the brain and also reduce microglial reactivity and motor deficit, in A2 adenosine receptor-dependent mechanism (Kozela et al., 2011; Mecha et al., 2013). Further to migration and morphological adaptation of immune cells, the release of proinflammatory cytokines is reduced in EAE animals treated with CBD (Rahimi et al., 2015; Elliott et al., 2018; Figure 2).

Another formulation of CBM can be used in the form of THC and CBD combination, due to pharmacological interaction and targets of both compounds in physiological systems. Even though isolated CBD and THC were effective in the clinical score reported for EAE mice, only the combination CBD/THC and isolated THC was able to induce long-term effectiveness in a CB1-dependent manner, which led to decrease in the disease progression (Moreno-Martet et al., 2015). Furthermore, a 1:1 combination of CBD/THC decreases the expression of Tumor Necrosis Factor-α (TNF-α) and increases Brain-Derived Neurotrophic Factor (BDNF) release (Zhou et al., 2019; Figure 2).

Considering that Cannabis extracts contain a large number of secondary metabolites from *Cannabis sativa*, the combined potential of these molecules could be important to induce a potentiated combined effect, called entourage effect. As an example, it has been shown that the terpene β-caryophyllene has the property of inhibiting T cell activity, along with reduced proinflammatory cytokine release, thus attenuating MS severity, inflammatory stress and spinal cord demyelination (Alberti et al., 2017).

Considering the diverse nature of MS symptoms, treatments currently available are not sufficient to mitigate the progression of this disorder and may lead to side effects, tolerance and toxicity. Thus, the search for new therapeutic approaches have intensified and phytocannabinoids have been demonstrated as important allies for MS treatment (Figure 2). In a parallel group, double-blind, randomized, placebo-controlled study, 160 MS patients were treated with oromucosal spray of CBM (2.7 mg THC and 2.5 mg CBD) during 6 weeks, with a mean dose of 2.5–120 mg of each daily. The Visual Analogue Scale (VAS) score was used to assess the primary outcome and results showed reduction of mean 74.36–48.89 in patients treated with Cannabis extract, significantly relevant when compared to placebo group (Wade et al., 2004). Later, the same group published a study to assess the safety and efficacy of this CBM, which included the evaluation of 137 MS patients refractory to standard drugs and described lack of efficacy in 42.3% of patients. For those who experienced improvement in clinical conditions, the amelioration of symptoms remained stable even after the acute phase and only mild to moderate adverse effects were reported (Wade et al., 2006).

The evaluation of CBM efficacy on spasticity of 189 patients in a double-blind study over 6 weeks, which showed positive change from baseline in the severity of spasticity measured by the 0–10 numerical rating scale (NRS), besides the Ashworth Scale and Motricity Index in muscles affected by spasticity, analyzed by intention-to-treat (ITT) (Collin C. et al., 2007). The group has performed a similar ITT analysis after NRS in a larger number of patients, refractory to anti-spasticity therapies, describing the efficiency of CBM in reducing treatment-resistant spasticity in treated subjects within the first 4 weeks of treatment (Collin et al., 2010).

In 2011, a multicenter study was conducted during 19 weeks to assess efficacy and safety of CBM in two phases. In phase A, which was single-blind and preliminary, subject responsiveness to CBM treatment has been evaluated for 4 weeks, and only those that showed at least 20% reduction in spasticity scores were allowed to proceed into phase B, a double-blind, randomized, parallel-group and placebo-controlled design, during 12 weeks. ITT analysis corroborated previous findings supporting CBM use for treating spasticity, besides positive outcome in Spasm Frequency Score, Sleep Disturbance NRS Patient, Carer and Clinician Global Impression of Change (Novotna et al., 2011). Posterior *post hoc* analysis of these data showed that results reported could be observed regardless of anti-spasticity pre-treatment history, accounting for consistent relief of symptoms and good tolerability (Haupts et al., 2016).

The phase 3 trial named MUSEC (MUltiple Sclerosis and Extract of Cannabis) consisted of a double-blind, randomized, placebo-controlled, multicenter study with 279 stable MS patients, initiated by a screening period, followed by a 2-week dose titration phase, then a 10-week maintenance period. Differently from the studies previously reported, capsules containing THC as the main cannabinoid were administered to patients twice daily. Results demonstrated relief of muscle stiffness, body pain, spasms and sleep quality after 12 weeks in approximately 29.4% of patients (Zajicek J. P. et al., 2012).

An observational, prospective, multicenter, and non-interventional study conducted in Germany, named MOVE 2, evaluated the response of patients with moderate to severe MS spasticity to CBM treatment. About 74.6% of patients showed relief in spasticity after 1 month, which was stable after 3 months in patients who could undergo follow-up analysis, in accordance with previous studies. Furthermore, improvements in sleep disorders and quality of life have also been reported, besides being well tolerated, once the most common adverse effects reported were dizziness, drowsiness, fatigue, nausea, and dry mouth (Flachenecker et al., 2014). To expand discoveries from the German experience, MOVE 2 design was applied to other European countries. Thus, results from Italy describe a similar phenomenon previously observed, once there at least 49% of patients showed stable improvement of NRS for spasticity after 3 months of treatment, whose main adverse events were dizziness and confusion, reported by 13% of patients in the 3-month follow-up period (Trojano and Vila, 2015).

More recently, the Belgian experience has also been reported in a retrospective study with 238 patients from 8 centers. Improvement in the MS spasticity NRS was significantly lower from 4 to 12 weeks when compared to baseline, which accounted for 73% of patients with improvement ≥ 20% in NRS. Additionally, the self-rated health-related life quality score (EuroQoL Visual Analog Scale) during treatment expressed a decreased disease burden perceived by patients, once 33% of them reported apparent amelioration of this aspect by 4 weeks and stable at 12 weeks (D’hooghe et al., 2021).

In summary, these data suggest that CBM composed by equal concentration of THC and CBD provides an efficient, well-tolerated and safe option to treat spasticity due to MS progression, besides being effective for amelioration of co-morbidities associated to quality of life of patients, such as sleep disorders, even though the progression of disease at neurodegenerative level is not completely assessed.

## Discussion

In this review, it was described how three of the most prevalent neurodegenerative diseases progress in terms of molecular, cellular and behavioral aspects, in addition to ponderations related to changes in life quality of patients and their caregivers. Despite the physiopathological and epidemiological differences among these disorders, some aspects are commonly shared, such as the absence of efficient and long-term therapeutic approaches to arrest disease progression, some primary symptoms and comorbidities, besides altered features in response to neuroinflammatory environment induced by pathological process occurring in the neural circuitry and unbalanced ECS.

Since the discovery of therapeutic applications of CBM and the description of possible mechanisms of action, they have been widely used in several countries, under particular regulatory procedures defined by each. Beyond anecdotal reports of symptom improvement, animal studies and clinical trials have been conducted in order to assess safety, efficacy and tolerability of these treatments in the context of neurodegenerative diseases, providing the scientific basis to guarantee plausible prescription of this therapeutic alternative.

In preclinical studies, conducted *in vitro* and *in vivo*, it has already been extensively demonstrated that phytocannabinoids and CBM can reverse altered cellular, molecular and behavioral aspects in AD, PD, and MS models. Considering AD, studies have reported reduction in astrocytic reactivity, neuroinflammation, memory loss, and cognitive scores, while in PD it was observed the reduction in cell death of dopaminergic neurons and neuroinflammation, associated with recovery of motor and cognitive ability in animals. Additionally, in MS, studies also describe reduced neuroinflammation, besides decreased infiltration of lymphocytes into the CNS and severity of spasticity.

Even though the clinical evidence to support the use of CBM for the treatment of MS are compelling, including the registration of pharmaceutical-grade products indicated for this purpose and a large number of patients assessed in clinical trials, the same is not true for AD and PD (Table 1 summarizes the main outcomes of clinical studies conducted with phytocannabinoids). Regarding the investigation on the use of cannabinoids and CBM in AD, there is currently one randomized placebo controlled clinical trial in progress and registered in the NIH clinical trials platform, to be performed in Eastern Virginia Medical School. This phase 2 study will evaluate the efficacy of a THC-enriched CBM over the agitation in 40 patients diagnosed with AD, as well as gain in life quality of both patients and caretakers (Okhravi and Sandoval, 2020 clinicaltrials.gov NCT04436081). For clinical studies involving phytocannabinoids and PD, there are currently two studies in progress registered in the NIH platform. One of the studies, a phase 2 randomized open-label study will be performed in the University Health Network with 15 patients in order to investigate safety and tolerability of three different CBM formulations, in addition to obtaining responses about frequency and severity of pain, sleep, dystonia and motor symptoms [Fox and Ropa (2018), clinicaltrials.gov NCT03639064]. A second study has been performed in Sheba Medical Center, an observational study with 100 patients in order to investigate the effect of CBM over non-motor symptoms developed in PD [Anis and Anis (2021), clinicaltrials.gov NCT051065014].

**TABLE 1:** Summary of main outcomes from clinical trials using CBM.

	Sample size	Dose	Main outcomes	References
Alzheimer’s disease	11 patients	1.5 mg THC 3 times a week during 3 weeks	No improvement in NPI	van den Elsen et al., 2015
			No changes in agitation, quality of life and daily activities	
	11 patients	2.5–5 mg THC twice a day during 4 weeks	Improvement in CGI	Shelef et al., 2016
			Reduced agitation, aggression, apathy, and sleep disorders	
			No modification in MMSE	
Parkinson’s disease	4 patients	75–300 mg CBD	Improvement in agitation, aggression, and sleep disorders	Chagas et al., 2014
	19 patients	0.034–0.25 mg/kg/day THC	No relief in parkinsonian symptoms and LID	Carroll et al., 2004
		0.017–0.125 mg/kg/day CBD	No changes in UPDRS score, PDQ-39, pain, and sleep quality	
Multiple sclerosis	160 patients	2.5–120 mg/day CBD + THC 1:1	Reduction in VAS score	Wade et al., 2004
			No significant adverse effects reported	
	137 patients	2.5–120 mg/day CBD + THC 1:1	Benefits from CBM are stable after acute phase	Wade et al., 2006
			No withdrawal syndrome in the majority of patients	
			Mild to moderate adverse effects	
	189 patients	Mean of approximately 23.5 mg/day CBD + THC 1:1 (9.4 sprays)	Reduction in NRS spasticity scores	Collin C. et al., 2007
			Global impression of improvement has been reported	
			Mild to moderate adverse effects reported	
	337 patients	2.5–55 mg/day CBD + THC 1:1 (1–22 sprays)	Improvement in spasticity NRS scores	Collin et al., 2010
			Mild to moderate adverse effects reported	
	538 patients	Mean of approximately 20.75 mg/day CBD + THC 1:1 (8.3 sprays)	Decrease in spasticity NRS scores	Novotna et al., 2011
			Reduced spasm frequency	
			Improvement in sleep disruption NRS	
			Treatment was well tolerated	
	279 patients	2.5–25 mg/day THC	Relief from muscle stiffness	Zajicek J. P. et al., 2012
			Relief from body pain	
			Urinary tract infection, head injury, and interstitial lung disease were considered treatment related by the investigator	
	276 patients	2.5–40 mg/day CBD + THC 1:1 (1–16 sprays)	Decrease in resistant MS spasticity	Flachenecker et al., 2014
			Reduced mean of NRS spasticity	
			Reduced sleeping disturbances	
			No safety concerns were raised	
	322 patients	2.5–30 mg/day CBD + THC 1:1 (1–12 sprays)	Decrease in spasticity NRS	Trojano and Vila, 2015
			Decrease in modified Ashworth score	
			Only 2 patients showed severe side effects (mental impairment and suicidal ideation)	
	238 patients	2.5–30 mg/day CBD + THC 1:1 (1–12 sprays)	Improvement in spasticity NRS	D’hooghe et al., 2021
			Increasing in mean EuroQoL Visual Analog Scale	
			Mild to moderate adverse effects reported	

Therefore, it is still necessary to enlarge the number of patients evaluated in clinical trials and a proper description of further benefits of CBM addition to treatment regimens, which could lead to wide application of these products to promote better life quality and mitigate at least partially the social and economic burden of such diseases.

## Author Contributions

YP-C, AA, AI, BF, RC, and PT wrote different sections of the review. BF designed the original figures from the manuscript. RM and LS provided intellectual assistance, reviewed, and corrected the manuscript. All authors performed the literature revision needed for this review.

## Conflict of Interest

The authors declare that the research was conducted in the absence of any commercial or financial relationships that could be construed as a potential conflict of interest.

## Publisher’s Note

All claims expressed in this article are solely those of the authors and do not necessarily represent those of their affiliated organizations, or those of the publisher, the editors and the reviewers. Any product that may be evaluated in this article, or claim that may be made by its manufacturer, is not guaranteed or endorsed by the publisher.

## Abbreviations

2-AG: 2-arachidonoylglycerol
2-MAGs: 2-monoacylglycerols
6-OHDA: 6-hydroxydopamine
A β: β -amyloid protein aggregates
AA: arachidonic acid
AD: Alzheimer’s Disease
AEA: anandamide
ApoE: apolipoprotein E
APP: amyloid precursor protein
BDNF: Brain-derived neurotrophic factor
CB1: cannabinoid receptor 1
CB2: cannabinoid receptor 2
CBD: cannabidiol
CBDV: cannabidivarin
CBG: cannabigerol
CBGV: cannabigerovarin
CBM: Cannabis-based medicine
CBN: Cannabinol
CGI: Clinical Global Impression
CNS: Central Nervous System
CREAE: chronic relapsing experimental autoimmune encephalomyelitis
DAGL α: diacylglycerol lipase alfa
DAGL β: diacylglycerol lipase beta
EAE: experimental autoimmune encephalomyelitis
ECS: endocannabinoid system
FAAH: fatty acid amide hydrolase
GMSCs: gingiva derived mesenchymal stem cells
GPCR: G-protein -coupled receptors
HSP: heat shock proteins
ITT: intention-to-treat
LID: levodopa-induced dyskinesia
MAGL: monoacylglycerol lipase
MHC II: major histocompatibility complex class II
MMP-2: metalloproteinase 2
MMP-9: metalloproteinase 9
MMSE: Mini Mental State Examination
MOVE 2: multicenter and non-interventional study conducted in Germany
MS: Multiple Sclerosis
MUSEC: Multiple Sclerosis and Extract of Cannabis
NAEs: N-acylethanolamines
NAPE-PLD: NAPE-specific phospholipase-D-like enzyme
NPI-: Neuropsychiatric Inventory scale
NRS: numerical rating scale
OEA: oleoylethanolamide
OPC: oligodendrocytes precursor cells
PD: Parkinson’s Disease
PEA: palmitoylethanolamide
PGC-1 α: PPAR γ coactivator 1 α
PPAR γ: Peroxisome Proliferator-Activated Receptor Gamma
PPMS: primary progressive multiple sclerosis
PSEN1: presenilin-1
PSEN2: presenilin-2
RNS-: reactive nitrogen species
ROS: reactive oxigen species
RRMS: relapsing-remitting multiple sclerosis
SOD1: Cu,Zn-Superoxide Dismutase
SPMS: secondary progressive multiple sclerosis
TFAM: mitochondrial transcription factor A
THC: Δ ^9^-tetrahydrocannabinol
THCV: Δ ^9^-tetrahydrocannabivarin
TNF- α: Tumor Necrosis Factor - α
TRPA1: transient receptor potential channel of ankyrin type-1
TRPM8: transient receptor potential cation channel subfamily M member 8
TRPV1: transient receptor potential channels of vanilloid type-1
UPDRS: Unified Parkinson’s Disease Rating Scale
VAS: Visual Analogue Scale
WHO: World Health Organization.
